# Secondary Female Anorgasmia in Patients with Obsessive Traits: A Study

**DOI:** 10.3390/bs14100953

**Published:** 2024-10-16

**Authors:** Giulio Perrotta, Stefano Eleuteri

**Affiliations:** 1 Istituto per lo Studio delle Psicoterapie (ISP), 00185 Rome, Italy; 2 Department of Developmental Psychology, Sapienza University Rome, 00166 Rome, Italy; stefano.eleuteri@uniroma1.it

**Keywords:** female orgasm, anorgasmia, obsessive–compulsive disorder, obsessive disorder, OCD

## Abstract

*Background*: In the literature, female anorgasmia (AO) is closely related to obsessive–compulsive disorder (OCD), but no publication has explored the role of individual obsessive traits that may also be typical of other disorders, assuming that repetitive thoughts and compulsions must necessarily correspond to an obsessive-type neurotic profile. It is worth investigating and assessing the specific weight of other morbid conditions involved, beyond OCD. *Materials and Methods*: This study was conducted during 2022–2023 by selecting 208 Italian clinical patients from private databases. They submitted to individual clinical interviews and two psychometric questionnaires (Perrotta Integrative Clinical Interviews 3, PICI-3-TA, and Perrotta Individual Sexual Matrix Questionnaire, PSM-Q). *Results*: Among the 208 patients (M: 39.05 years), divided into subgroups by age and obsessive symptomatology, no significant differences were found between the subgroups in the comparisons of the data obtained from the administration of the PSM-Q. *Conclusions*: This study confirms that it is not OCD that correlates with OA but obsessive traits, which are also common to other psychopathological disorders, such as bipolar disorder, borderline disorder, manic, and psychotic disorders and, therefore, in psychotherapy, it is necessary to intervene with a clinical approach that has in mind the patient’s psychopathological personality picture and the causes that originated or reinforced OA.

## 1. Introduction

An orgasm is an intended and overwhelming experience [1]. It represents, in the collective imagination, the successful conclusion of solitary (by masturbation) or shared (by preliminary and/or full intercourse) sexual activity [2]. It can be single or multiple and can be achieved by clitoral, vaginal, vagino-cervical, and (more rarely) anal stimulation [3]. Specifically, for a vaginal orgasm, penetration is required in each case and is the product of the stimulation of the vaginal walls, while in a clitoral orgasm there is stimulation, by friction, exclusively of the clitoris and it is from this that the sensation of pleasure originates. Finally, in an anal orgasm, the sensation produced depends on several factors, predominantly fantasy and subjective arousal, the sensation of pain/pressure, and the rubbing of the walls adjacent to the rectum. It is still debated whether a cervical orgasm is autonomous or still depends on vaginal penetration using the male sex organ or a physical object of significant size [3]. An orgasm represents the third phase of pleasure attainment, which begins with arousal (first phase), evolves into a pleasure peak or plateau (second phase), continues with an orgasm (third phase), and concludes with relaxation or resolution (fourth phase) [4]. 

With dysfunctional conditions, however, some women may experience anorgasmia, which was formerly referred to as frigidity, as defined in diagnostic manuals, such as the *International Classification of Diseases*, 11th Revision (ICD-11); the *Diagnostic and Statistical Manual of Mental Disorders*, latest revised edition (DSM-5TR); and in international guidelines [5,6]. According to the statistics, depending on age and other variables, it is estimated that between 4% and 12% of women not in aa relationship, and up to 20% of women in a relationship are sexually anorgasmic, with a peak of 40% to 50% of women faking an orgasm on more than one occasion during intercourse with their partners [6,7]. 

Today, AO is defined in the literature as the absence of orgasm (or the achievement of an orgasm with high difficulty) and, thus, the more or less complete interruption of the sexual pleasure cycle [8]. In the past, cases of delayed orgasms, i.e., conditions of extreme difficulty in reaching orgasm, were also mentioned in anorgasmia; today, however, it is preferred to separate the two categories [9,10]. 

Anorgasmia (AO), therefore, may be considered primary if a woman has never experienced an orgasm in her life, while it may be considered secondary when the absence is acquired after having already experienced it [11]. Several etiological causes [12,13,14,15,16,17,18,19,20,21,22,23,24,25,26,27,28,29] may affect this disorder, often in combination; the main ones that have been identified in the literature are (a) anatomic and pathophysiologic factors, such as occurs in clitoral priaprism; as a result of physical damage after physical injury or after childbirth; as a result of bacterial, fungal, and/or viral infections that result in painful female coitus; or as a result of disabling physical conditions, such as multiple sclerosis, spinal cord injuries, bladder parenchymal contraction deficits, and vulvovaginal atrophy; (b) substance-induced factors, such as occurs in an altered state of alcohol or drug consumption abuse (as in the case of cocaine, cannabinoids, and opiates) and/or pharmacological substances (as in the case of using antidepressants and anxiolytics, such as alprazolam, fluoxetine, fluvoxamine, and clomipramine), capable of affecting female hormonal processes (including ovulation); and (c) psychological factors, such as occurs in cases of anxiety, mood swings, depressive states, obsessive states and, more generally, psychological trauma of a sexual nature. 

It is, moreover, well known from the literature that sexual disorders (among which AO is included) are very common problems in psychiatric patients [30], both because of the interference of dysfunctional symptoms in daily life and because of the side effects of some medications used in psychiatry and neurology [31]. For example, bipolar disorder significantly affects quality of life and increases the risk of sexual dysfunction, as in the manic phase when there is a disproportionate increase in libido, with which risky or legally impermissible sexual behaviors are associated, while in the depressive phase, there is a marked reduction in libido, leading to the sexual dissatisfaction/frustration of the same subject and partner. Again, anxiety disorders result in decreased sexual desire and an aversion to sexual acts, while with psychotic disorders, in particular AO, decreased libido (and, in men, ejaculatory delay and difficulty in maintaining penile rigidity), vaginal dryness, vaginismus, and dyspareunia are frequent. Finally, in patients with eating disorders, the libido decreases as does sexual activity, and the level of sexual satisfaction decreases. However, the highest rate of frequent AO is related to patients with obsessive–compulsive disorder (OCD). 

The neurobiological nature of anorgasmic disorder seems to have been confirmed by neuroimaging and electrical stimulation studies, but there is still no clear position in the literature on this [32,33,34]. Specifically, the areas that appear to be involved in the mechanism of AO are the hypothalamus, periaqueductal gray of the midbrain, hippocampus, and cerebellum [35], but also other areas, such as the sensory, motor, reward, frontal cortical, and brainstem regions (e.g., nucleus accumbens, insula, anterior cingulate cortex, orbitofrontal cortex, operculum, right angular gyrus, paracentral lobule, amygdala, hypothalamus, ventral tegmental area, and dorsal raphe) [36], as is the case in OCD, which involves the insula, anterior cingulate cortex, bilateral caudate nucleus, left superior temporal gyrus, precuneus, parahippocampal regions, and dorsolateral prefrontal and left orbitofrontal cortex [37,38,39].

No studies or reviews have emerged from the literature that address the issue of the active role of obsessive traits outside of obsessive–compulsive disorder, although these traits are also characteristic of other psychiatric disorders, as already indicated. Investigating the role of these dysfunctional traits beyond classic obsessive–compulsive disorder could improve the understanding of these patients’ symptoms.

The present research, examining patients with OCD, focuses precisely on the psychological factors in AO, aiming to frame it more systematically around its psychological and symptomatological profile. One wonders whether a difference between OCD and obsessive traits (OT) is also typical of other psychopathological disorders, and whether these morbid conditions have significant differences.

The main objective of the present research, based on the results from the literature, is to demonstrate that obsessive traits, regardless of the specific psychopathology (whether neurotic, dramatic, or psychotic), are involved in the mechanism of OA in cisgender woman. The secondary objectives are as follows: (1) Is OA related to singular obsessive traits or only to OCD? (2) Is OA a morbid condition that exists pre-detached from obsessive traits? (3) Is it possible to determine a severity scale of OA based on obsessive symptoms? (4) Can treating obsessive traits with a clinical approach reduce the negative impact of OA on patients?

## 2. Materials and Methods

**Figure 1:**
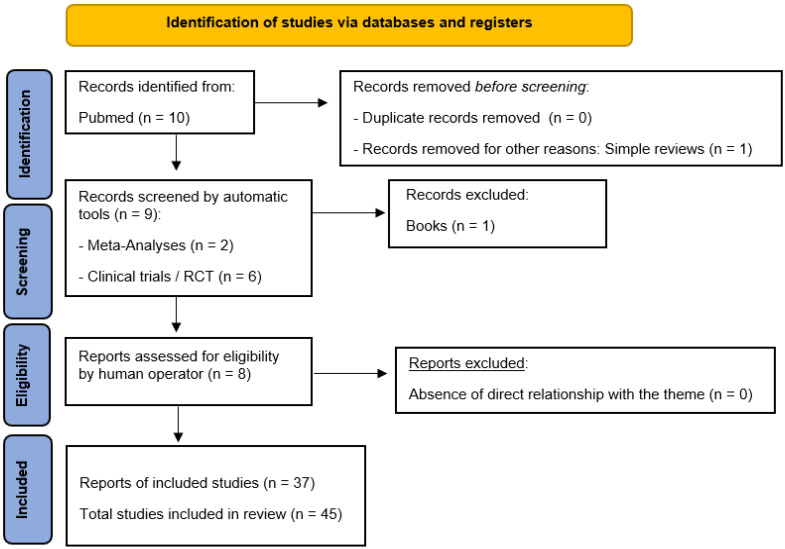
PRISMA flow diagram template [40].

With a specific search on PubMed, using the terminological indicators “obsessive-compulsive disorder” and “anorgasmia”, 45 useful results were selected. No limit was placed on the year of publication, until the completion date of this study (February 2024) (Figure 1).

The method consists of conducting clinical interviews of female anorgasmic subjects based on the Perrotta Human Emotions Model 2 (PHEM-2) [41], first administering the Perrotta Integrative Clinical Interviews-3 (PICI-3) [42] to identify the functional and dysfunctional personality traits (to confirm the previous psychopathological diagnosis characterized by obsessive traits), and then administering the Perrotta Individual Sexual Matrix Questionnaire (PSM-Q) [43] for the identification of individual sexual matrix characteristics, to assess the symptom profile related to anorgasmia (relative to the main reasons that would account for anorgasmia). Specifically, (a) the Perrotta Human Emotions Model 2 (PHEM-2) is a model that reorganizes the structural and functional structure of emotions, according to a precise scheme using 226 different emotional trajectories, linking the different emotional particles into columns and rows dedicated to the adaptive mode, emotional and feeling state, and emotional reactions and their consequences; (b) the Perrotta Integrative Clinical Interviews-3 (PICI-3) is a validated polyquestionnaire, which individuates functional and dysfunctional personality traits, performing a rationalization of dysfunctional processes using 25 different personality categories, and 18 for the functional ones; (c) the Perrotta Individual Sexual Matrix Questionnaire (PSM-Q) is a polyquestionnaire with a survey function, to collect information related to the individual’s sexual matrix, according to the correspondent model, and investigates the sexual dimension of both the functional and dysfunctional parts. Connected to the PSM-Q are 3 questionnaires with clinical functions and a ratings scale, which are in the validation phase; in this study they were not used as they are not necessary and not yet validated. 

The phases of this research were divided as follows: (1) the selection of the population sample, according to the parameters indicated in the following paragraph; (2) clinical interviews, based on PHEM-2; (3) the administration of the psychometric tests, such as the PICI-3 for the study of functional and dysfunctional personality traits and the PSM-Q for the identification of individual sexual matrix characteristics; and (4) data processing following the administration and comparison of the data obtained.

## 3. Setting and Participants

**Figure 2:**
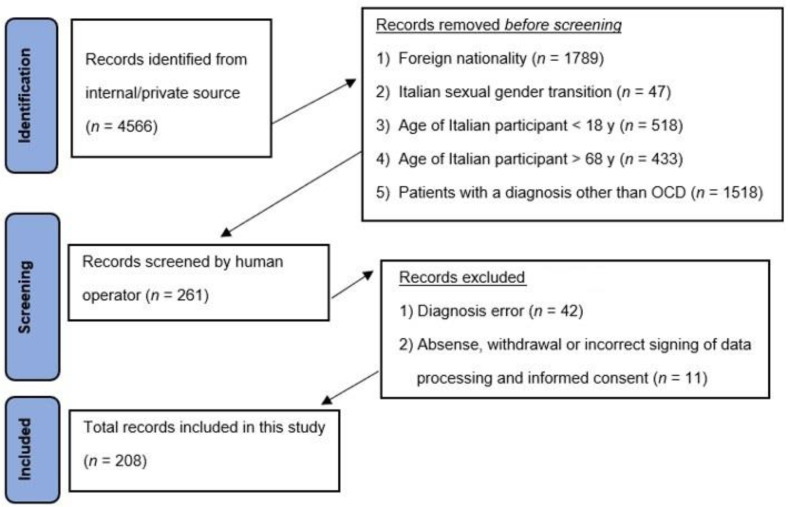
Criteria for selection of population sample.

The inclusion criteria were as follows: (1) age between 18 and 68 years; (2) defined sexual gender (female) and Italian nationality; (3) cisgender; (4) a diagnosis of OCD at least 12 months prior, with or without psychotherapeutic and/or pharmacological treatment; and (5) a diagnosis of AO secondary to OCD (subsequently acquired) and its persistence for at least 12 months (the patient reports anorgasmia or an inability to achieve orgasm through nonpersonal intercourse or masturbation), with or without psychotherapeutic and/or pharmacological treatment. The exclusion criteria were as follows: (1) age under 18 or over 68 years old; (2) no defined sexual gender; (3) no Italian nationality; (4) psychiatric diagnosis in the absence of AO, regardless of the duration and persistence of the disorder; (5) a diagnosis of primary OA or secondary OA with the achievement of orgasm by an individual sexual act (masturbation), with the person’s achievement time and manner; and (6) the absence, withdrawal, or incorrect signing of the data processing and informed consent (Figure 2).

The setting chosen, taking into account the protracted pandemic period of COVID-19 and the geographic residence of the participants (across the Italian territory), was the online platform Skype and video calls via WhatsApp, both for the clinical interview and for the administration of the questionnaire, using a private database of patients who agreed to at least one initial cycle of psychotherapy (three sessions minimum). The present research was carried out from January 2022 to December 2023, and the use of online platforms did not generate practical difficulties in conducting the clinical interviews or the administration of the psychometric instruments, making the data retrieval work more functional and smooth than the potential choice of convening all the participants in person, who nevertheless participated with enthusiasm and interest. 

The selected population sample that met the inclusion and exclusion requirements consisted of 208 participants (Table 1).

## 4. Results 

After selecting the chosen population sample (first stage) according to the inclusion and exclusion criteria, clinical interviews (second stage) were conducted, from which the following data emerged:

1. There were 261 subjects who underwent PICI-3 [42] for dysfunctional traits, to check whether the previous diagnosis of obsessive–compulsive disorder was confirmed or not, but 42 (16.1%) were excluded due to a misdiagnosis or change in diagnosis, and 11 (4.2%) due to legal reasons, arriving at a total of 208 confirmed subjects. Of the 208 participants who completed this study, exactly half (104/208, 50%) were found to have a personality profile characterized by at least five dysfunctional personality traits of the obsessive type, while the other half had at least five dysfunctional personality traits of other types (in particular and in descending order, bipolar disorder and psychotic disorders, then masochistic disorder, depressive disorder, anxiety disorder, dipendant disorder, and manic disorder), with the obsessive traits in a secondary or tertiary position.
2. Using strategic language and PHEM-2 during the interview [41], the totality of the final selected population sample (208/208, 100%), both in the first and second groups, showed a complete distress orientation, expressing feelings such as anger, frustration, fear, and disappointment, with the presence of stressogenic events typical of their clinical condition of AO. Specifically, independently of the primary diagnosis, the totality of the sample reported that the obsessive symptomatology related to the inability to achieve orgasm, whether alone during masturbation or in a single or paired penetrative act, was constant, generating high levels of expectation and then disillusionment, which, as in a self-fulfilling prophecy, returns to the impossibility of achieving orgasm, resetting the libido. There was no statistically significant difference in this regard between the two groups.
3. The administration of the Perrotta Individual Sexual Matrix Questionnaire (PSM-Q) [43] found 100% positivity in the overall sample (208/208) regarding the persistence of the anorgasmic condition, in both groups with no statistically significant differences. It imputed one-fifth (42/208) of the cases to be the result of moderate-to-severe psychological disorders (with a slight quantitative tendency toward the obsessive–compulsive disorder group, 25/42, 60%); this was followed by feelings of guilt and shame, excessive control, distrust of the relationship as a result of facts or events that produced disappointment and were able to negatively affect mutual esteem and respect, lack of confidence with the partner and sex, and physical ailments. Specific phobias seemed to be the least impactful category, reflecting the fact that the phobic component often combines with the obsessive component, reinforcing each other.

Using IBS’s software application for statistical analysis (Statistical Package for Social Science, SPSS, version 29.0), descriptive, frequency, and mean comparison analyses (t-test for independent data) were performed; by first comparing the OCD-pure group (subjects with OCD as the primary diagnosis) with the variables in the PSM-Q about the main reasons that would justify anorgasmia, and then with the OCD-comb group (subjects with OCD as a secondary diagnosis), again with the variables in the PSM-Q about the main reasons that would justify anorgasmia, the statistical analysis showed that there were no statistically significant differences between the groups (*p* > 0.05). The data have been corrected for error rates, as per the statistics compiled (Table 2). 

Even if we correlate the anorgasmic condition with the individual relevant psychopathological traits in the selected population, it emerges that the traits are not statistically significant (*p* > 0.05). This demonstrates and confirms the fact that it is not the obsessive condition that determines the symptomatology described in the history, and thus the reason for the discomfort experienced, but it is the presence of the individual obsessive traits that interrupt the pleasure circuit and determine the repetitive and negative thoughts underlying the psychopathological condition and the dysfunctional personality tendencies, which need not necessarily be traced back to OCD (Table 3).

## 5. Discussions and Limits 

The present study has shown that it is obsessive traits and not obsessive compulsive disorder that correlate with female secondary anorgasmia, as presenting obsessive traits in one’s personality profile does not necessarily mean one has the obsessive disorder, as these traits are also present in other disorders, such as in the anxiety spectrum, psychotic spectrum, bipolar disorder, manic disorder, depressive disorder, masochism profile, and dependent disorder, according to the PICI model, and therefore assessing the personality profile is prodromal to the diagnosis of specific disorder. The obsessive traits among these patients, both in the primary hypothesis (OCD as a primary disorder) and secondary hypothesis (OCD as a secondary disorder), impact them negatively, fostering those anxiogenic and anxious processes capable of decreasing or eliminating libido, before and/or during intercourse, effectively preventing the conclusion of female intercourse. There is a profound difference between obsessive–compulsive disorder and simple obsessive traits (which are only a part of OCD), but this research has shown precisely that the clinical focus should be on the management of obsessive traits, regardless of the diagnosis status, and the literature suggests that cognitive–behavioral and strategic approaches are among the most effective [44,45]. Thus, the statistical data obtained show that there are no statistically significant differences (*p* > 0.05) and, therefore, there is no statistical evidence (i.e., significance) that the two groups (“OCD_pure”-“OCD_comb”), relative to the outcome of the matrix, are different. This effectively reinforces, with a counterfactual mechanism, the hypothesis that the central theme must be the obsessive traits themselves and not just OCD. The confirmation of statistical significance (*p* < 0.01) emerges when comparing the two groups concerning the individual obsessive traits.

The present research has succeeded in demonstrating that the specific weight of the individual obsessive traits is greater than the diagnosis of a specific disorder (including OCD) itself, in patients who present with OA, and therefore attention should be paid first to the individual traits and the personality profile, with respect to its structural and functional components. Thus, it is confirmed that obsessive traits, regardless of the specific psychopathology (neurotic, dramatic, or psychotic), are involved in the mechanism of OA. The data, in addition, allow us to answer all the secondary objectives as well, including that (1) OA is related to singular obsessive traits and not only to OCD, which only represents the pathology with the highest obsessive incidence in the neurotic-type psychiatric picture; (2) OA is a morbid condition markedly characterized by obsessive traits; (3) based on the symptoms described in the medical histories and during the clinical interviews, it is possible to determine the severity of the impact of obsessive traits on OA and the severity of OA as a sexual disorder using validated psychometric tests; (4) by identifying the causes that explain the existence of OA, through the use of the PSM-Q, it is possible to intervene with a clinical approach to the condition, working on an individual’s obsessive traits and disregarding the underlying psychopathological condition. 

This study’s most significant finding is that obsessive traits, regardless of the underlying psychopathology, have a direct correlation with OA. This discovery shifts the focus from diagnosing a singular disorder to managing the individual personality traits that contribute to sexual dysfunction. 

The present study, however, has both structural and functional limitations that, in the opinion of the authors, do not invalidate the quality of the results obtained, but should be taken into consideration in future research to avoid analytical bias. Structurally, the design of this study did not include the use of neuroimaging data that could have provided detailed feedback about the anorgasmia reported by the patients; however, it was felt that this lack did not invalidate the quality of the work performed, and that this specification could be integrated into a second and future manuscript to reinforce or refute the preliminary outcome. Functionally, this study recruited 208 adult female subjects who had been diagnosed with secondary and persistent OA for at least 12 months, but the hypothesis of primary anorgasmia and the difficulty in achieving orgasm by a full act were not taken into account, nor was it taken into account whether or not these selected subjects had undergone courses of psychotherapy or had used one or more drug therapies. These apparent shortcomings were assessed as not affecting the outcomes of this study, as they pertain to a later clinical phase that will be incorporated into the next planned study; this preliminary study was focused exclusively on female subjects diagnosed with secondary and persistent OA, as the focus of this work was on the dysfunctional assessment of obsessive traits for a possible diagnoses status and, therefore, this selected target appeared to be the most appropriate for conducting this analysis. 

In the future, therefore, the direction of study will be to integrate current knowledge with neuroimaging outcomes and prescribed or to-be-prescribed therapies to have as broad and detailed a systematic picture as possible.

**Key Points**. 1. Female anorgasmia is related to obsessive–compulsive disorder through obsessive traits, which characterize the disorder. 2. Obsessive traits are also present in other psychopathological disorders, and therefore a personality study is needed to define the real dysfunctional extent of these traits. 3. It is more important to focus clinical action in psychotherapy on the study of individual obsessive traits, as they may not be exclusively the representation of obsessive–compulsive disorder.

## 6. Conclusions

In conclusion, the present study was able to confirm that the clinical focus on OA should be more on the individual obsessive traits and not only on the stated disorder, such as OCD (mainly), bipolar disorder, borderline disorder, manic, or psychotic disorders (other psychiatric disorders where the obsessive component is markedly significant). It is the obsessive traits that are related to OA and that maintain the dysfunctionality, regardless of the specific pathological personality profile. In psychotherapy, the early and targeted intervention on the obsessive traits, while having a clear personality profile of the subject and the causes that triggered or reinforced the OA, can promote a better resolution of the clinical problem, also because of the administration of a more targeted psychopharmacological therapy. 

## Figures and Tables

**Table 1:** *Population sample (clinical variables)*. Total Age (18–68 y): Age_18–27, Age_28–37, Age_38–47, Age_48–57, and Age_58–68. OCD_pure (or PICI-3_0_obs): all cases of OCD as the principal diagnosis according to the PICI model. OCD_comb: all cases of OCD as the secondary diagnosis according to the PICI model. PICI-3_1_anx: all cases of OCD as the secondary diagnosis and anxiety disorder as the primary diagnosis. PICI-3_2_maniac: all cases of OCD as the secondary diagnosis and maniac disorder as the primary diagnosis. PICI-3_3_depres: all cases of OCD as the secondary diagnosis and depressive disorder as the primary diagnosis. PICI-3_4_bipol: all cases of OCD as the secondary diagnosis and bipolar disorder as the primary diagnosis. PICI-3_5_dep: all cases of OCD as the secondary diagnosis and dependence disorder as the primary diagnosis. PICI-3_6_masoc: all cases of OCD as the secondary diagnosis and masochistic disorder as the primary diagnosis. PICI-3_7_psicot: all cases of OCD as the secondary diagnosis and psychotic disorders as the primary diagnosis. PSM-Q_1_control: the subject reports that the cause of anorgasmia is primarily an excessive tendency to control and brood over sex-related facts or events. PSM-Q_2_distrust: the subject reports that the cause of anorgasmia is primarily a distrust of the relationship as a result of facts or events that produced disappointment and were capable of negatively impacting mutual esteem and respect, excluding omissions, lies, and betrayals of a sexual nature. PSM-Q_3_low_confidence: the subject reports that the cause of anorgasmia is primarily distrust in the relationship as a result of facts or events that produced disappointment and were capable of negatively impacting trust, excluding omissions, lies, and betrayals of a sexual nature. PSM-Q_4_guilt/shame: the subject reports that the causes of anorgasmia are mainly feelings of guilt and shame relative to the sexual behavioral sphere. PSM-Q_5_physical_dysfunctions: the subject reports that the cause of anorgasmia is primarily one or more physical disorders. PSM-Q_6_mental_dysfunctions: the subject reports that the cause of anorgasmia is primarily one or more mental disorders. PSM-Q_7_specific_phobias: the subject reports that the cause of anorgasmia is primarily one or more specific phobias (e.g., fear of contracting sexually transmitted diseases, fear of unwanted pregnancy, fear of disappointing someone with the sexual act). PSM-Q_8_no_attraction: the subject reports that the cause of anorgasmia is mainly the total or partial absence of physical attraction or lack of passion. PSM-Q_9_childhood_sexual_abuse: the subject reports that the cause of anorgasmia is primarily abuse of a sexual nature suffered in childhood and/or preadolescence. PSM-Q_10_stressful life: the subject reports that the cause of anorgasmia is mainly stressors brought about by their work and/or family lifestyle. PSM-Q_11_sexual concerns: the subject reports that the cause of anorgasmia is mainly the different stance on sexual dynamics (e.g., paraphilias, improper or inappropriate conduct that the partner insistently shares or would like to share despite advice to the contrary). PSM-Q_12_unresolved_couple_problems: the subject reports that the cause of anorgasmia is mainly sexual omissions, lies and betrayals, or well-founded suspicions of them.

Variable	N	M ± SD or %
Total Age (18–68 y)	208	39.05 ± 14.62
Age_18–27 y	65	31.2%
Age_28–37 y	35	16.8%
Age_38–47 y	41	19.7%
Age_48–57 y	39	18.8%
Age_58–68 y	28	13.5%
Patients with a clinical diagnosis of OCD without comorbidities	104	50.0%
Patients with a clinical diagnosis of OCD with comorbidities	104	50.0%
PICI-3_1_anxiety disorder	14	13.5%
PICI-3_2_maniac disorder	8	7.7%
PICI-3_3_depressive disorder	15	14.4%
PICI-3_4_bipolal disorder	20	19.2%
PICI-3_5_dependence disorder	9	8.7%
PICI-3_6_masochistic disorder	18	17.3%
PICI-3_7_psychopathic disorder	20	19.2%
PSM-Q_1_control	20	9.6%
PSM-Q_2_distrust	20	9.6%
PSM-Q_3_low_confidence	19	9.1%
PSM-Q_4_guilt/shame	30	14.4%
PSM-Q_5_physical_dysfunctions	20	9.6%
PSM-Q_6_mental_dysfunctions	39	18.8%
PSM-Q_7_specific_phobias	9	4.3%
PSM-Q_8_no_attraction	10	4.8%
PSM-Q_9_childhood_sexual_abuse	11	5.4%
PSM-Q_10_stressful life	10	4.8%
PSM-Q_11_sexual_concerns	10	4.8%
PSM-Q_12_unresolved_couple_problems	10	4.8%

**Table 2:** *S.P.S.S. with a t-test for comparison.* OCD-pure (subjects with OCD as the primary diagnosis). OCD-comb (subjects with OCD as a secondary diagnosis). PSM-Q_1_control: the subject reports that the cause of anorgasmia is primarily an excessive tendency to control and brood over sex-related facts or events. PSM-Q_2_distrust: the subject reports that the cause of anorgasmia is primarily distrust of the relationship as a result of facts or events that produced disappointment and were capable of negatively impacting mutual esteem and respect, excluding omissions, lies, and betrayals of a sexual nature. PSM-Q_3_low_confidence: the subject reports that the cause of anorgasmia is primarily distrust in the relationship as a result of facts or events that produced disappointment and were capable of negatively impacting trust, excluding omissions, lies, and betrayals of a sexual nature. PSM-Q_4_guilt/shame: the subject reports that the causes of anorgasmia are mainly feelings of guilt and shame relative to the sexual behavioral sphere. PSM-Q_5_physical_dysfunctions: the subject reports that the cause of anorgasmia is primarily one or more physical disorders. PSM-Q_6_mental_ dysfunctions: the subject reports that the cause of anorgasmia is primarily one or more mental disorders. PSM-Q_7_specific_phobias: the subject reports that the cause of anorgasmia is primarily one or more specific phobias (e.g., fear of contracting sexually transmitted diseases, fear of unwanted pregnancy, fear of disappointing someone with the sexual act). PSM-Q_8_no_attraction: the subject reports that the cause of anorgasmia is mainly the total or partial absence of physical attraction or lack of passion. PSM-Q_9_childhood_sexual_abuse: the subject reports that the cause of anorgasmia is primarily abuse of a sexual nature suffered in childhood and/or preadolescence. PSM-Q_10_stressful life: the subject reports that the cause of anorgasmia is mainly the stressors brought about by their work and/or family lifestyle. PSM-Q_11_sexual concerns: the subject reports that the cause of anorgasmia is mainly the different stance on sexual dynamics (e.g., paraphilias, improper or inappropriate conduct that the partner insistently shares or would like to share despite advice to the contrary). PSM-Q_12_unresolved_couple_problems: the subject reports that the cause of anorgasmia is mainly sexual omissions, lies and betrayals, or well-founded suspicions of them. 95% CI (L): maximum confidence interval value; 95% CI (U): minimum value of confidence interval; df: degrees of freedom; *p*: significance level.

Variable Comparison	F	*t*	CI (min)	CI (max)	*p*
OCD_pure—PSM-Q_1_control	0.88	0.49	−0.06	0.10	0.642
OCD_pure—PSM-Q_2_distrust	0.00	0.00	−0.08	0.08	1.000
OCD_pure—PSM-Q_3_low_confidence	0.23	−0.24	−0.09	0.07	0.811
OCD_pure—PSM-Q_4_guilt/shame	0.62	0.39	−0.08	0.12	0.702
OCD_pure—PSM-Q_5_physical_dysfunctions	0.23	0.24	−0.07	0.09	0.814
OCD_pure—PSM-Q_6_mental_dysfunctions	1.13	−0.53	−0.14	0.08	0.606
OCD_pure—PSM-Q_7_specific_phobias	0.46	−0.34	−0.06	0.05	0.730
OCD_pure—PSM-Q_8_no_attraction	1.68	0.65	−0.04	0.08	0.526
OCD_pure—PSM-Q_9_childhood_sexual_abuse	0.00	0.00	−0.06	0.06	1.000
OCD_pure—PSM-Q_10_stressful life	1.68	0.60	−0.08	0.04	0.520
OCD_pure—PSM-Q_11_sexual_concerns	1.68	0.65	−0.08	0.04	0.520
OCD_pure—PSM-Q_12_unresolved_couple_problems	1.68	0.65	−0.04	0.08	0.520
OCD_comb—PSM-Q_1_control	0.88	−0.47	−0.10	0.06	0.640
OCD_comb—PSM-Q_2_distrust	0.00	0.00	−0.08	0.08	1.000
OCD_comb—PSM-Q_3_low_confidence	0.23	0.24	−0.07	0.09	0.811
OCD_comb—PSM-Q_4_guilt/shame	0.62	−0.39	−0.12	0.08	0.695
OCD_comb—PSM-Q_5_physical_dysfunctions	0.23	−0.24	−0.09	0.07	0.811
OCD_comb—PSM-Q_6_mental_dysfunctions	1.13	0.53	−0.08	0.14	0.596
OCD_comb—PSM-Q_7_specific_phobias	0.46	0.34	−0.05	0.06	0.735
OCD_comb—PSM-Q_8_no_attraction	1.68	−0.65	−0.08	0.04	0.519
OCD_comb—PSM-Q_9_childhood_sexual_abuse	0.00	0.00	−0.06	0.06	1.000
OCD_comb—PSM-Q_10_stressful life	1.68	0.65	−0.04	0.08	0.519
OCD_comb—PSM-Q_11_sexual_concerns	1.68	0.65	−0.04	0.08	0.519
OCD_comb—PSM-Q_12_unresolved_couple_problems	1.68	0.65	−0.08	0.04	0.519

**Table 3:** *S.P.S.S. with t-test for comparison.* OCD-pure (subjects with OCD as the primary diagnosis). OCD-comb (subjects with OCD as a secondary diagnosis). PICI-3_1_anx: all cases of OCD as a secondary diagnosis and anxiety disorder as the primary diagnosis. PICI-3_2_maniac: all cases of OCD as a secondary diagnosis and maniac disorder as the primary diagnosis. PICI-3_3_depres: all cases of OCD as a secondary diagnosis and depressive disorder as the primary diagnosis. PICI-3_4_bipol: all cases of OCD as a secondary diagnosis and bipolar disorder as the primary diagnosis. PICI-3_5_dep: all cases of OCD as a secondary diagnosis and dependence disorder as the primary diagnosis. PICI-3_6_masoc: all cases of OCD as a secondary diagnosis and masochistic disorder as the primary diagnosis. PICI-3_7_psicot: all cases of OCD as a secondary diagnosis and psychotic disorders as the primary diagnosis. 95% CI (L): maximum confidence interval value; 95% CI (U): minimum value of confidence interval; df: degrees of freedom; *p*: significance level.

Variable Comparison	F	*t*	CI (min)	CI (max)	*p*
OCD_pure—PICI-3_1_anx	0.000	0.000	−0.073	0.073	1.000
OCD_pure—PICI-3_2_maniac	0.000	0.000	−0.073	0.073	1.000
OCD_pure—PICI-3_3_depres	0.000	0.000	−0.073	0.073	1.000
OCD_pure—PICI-3_4_bipol	0.000	0.000	−0.069	0.069	1.000
OCD_pure—PICI-3_5_dep	1.222	0.551	−0.050	0.088	0.582
OCD_pure—PICI-3_6_masoc	0.000	0.000	−0.069	0.069	1.000
OCD_pure—PICI-3_7_psicot	0.000	0.000	−0.069	0.069	1.000
OCD_comb—PICI-3_1_anx	0.000	0.000	−0.073	0.073	1.000
OCD_comb—PICI-3_2_maniac	0.000	0.000	−0.073	0.073	1.000
OCD_comb—PICI-3_3_depres	0.000	0.000	−0.073	0.073	1.000
OCD_comb—PICI-3_4_bipol	0.000	0.000	−0.069	0.069	1.000
OCD_comb—PICI-3_5_dep	1.222	−0.551	−0.088	0.050	0.582
OCD_comb—PICI-3_6_masoc	0.000	0.000	−0.069	0.069	1.000
OCD_comb—PICI-3_7_psycot	0.000	0.000	−0.069	0.069	1.000

## Data Availability

The subjects who participated in this study requested and obtained that GP be the sole examiner during the therapeutic sessions and that all other authors be aware of the participants’ data in an exclusively anonymous form.

## Abbreviations

Female anorgasmia (AO). Obsessive–compulsive disorder (OCD). Perrotta Integrative Clinical Interviews 3 (PICI-3). Perrotta Human Emotions Model 2 (PHEM-2). Perrotta Individual Sexual Matrix Questionnaire (PSM-Q).
